# Interval Type-3 Fuzzy Adaptation of the Bee Colony Optimization Algorithm for Optimal Fuzzy Control of an Autonomous Mobile Robot

**DOI:** 10.3390/mi13091490

**Published:** 2022-09-07

**Authors:** Leticia Amador-Angulo, Oscar Castillo, Patricia Melin, Juan R. Castro

**Affiliations:** 1 Division of Graduate Studies, Tijuana Institute of Technology, TecNM, Tijuana 22414, Mexico; 2 School of Engineering, UABC University, Tijuana 22500, Mexico

**Keywords:** interval type-3 fuzzy logic, intelligent controllers, mobile robot, disturbance, uncertainty

## Abstract

In this study, the first goal is achieving a hybrid approach composed by an Interval Type-3 Fuzzy Logic System (IT3FLS) for the dynamic adaptation of α and β parameters of Bee Colony Optimization (BCO) algorithm. The second goal is, based on BCO, to find the best partition of the membership functions (MFs) of a Fuzzy Controller (FC) for trajectory tracking in an Autonomous Mobile Robot (AMR). A comparative with different types of Fuzzy Systems, such as Fuzzy BCO with Type-1 Fuzzy Logic System (FBCO-T1FLS), Fuzzy BCO with Interval Type-2 Fuzzy Logic System (FBCO-IT2FLS) and Fuzzy BCO with Generalized Type-2 Fuzzy Logic System (FBCO-GT2FLS) is analyzed. A disturbance is added to verify if the FBCO-IT3FLS performance is better when the uncertainty is present. Several performance indices are used; RMSE, MSE and some metrics of control such as, ITAE, IAE, ISE and ITSE to measure the controller’s performance. The experiments show excellent results using FBCO-IT3FLS and are better than FBCO-GT2FLS, FBCO-IT2FLS and FBCO-T1FLS in the adaptation of α and β parameters.

## 1. Introduction

In the last three years, a new methodology, to handle high levels of uncertainty in complex problems, has been presented through the study of IT3FLSs. At the present time, new problems have appeared that need control under uncertainty, and various research works had focused their contributions on controlling systems with better stabilization by analyzing the uncertainty as precisely as possible, such as; Castillo et al., in [1], study the theory and design of the IT3FLS; Castillo et al., in [2], propose a methodology for building IT3FLSs utilizing the granularity concept; Castillo et al., in [3], analyzed an IT3FLS in the implementation of Time Series Prediction; Sing et al., in [4], study an approach to the design of IT3FLSs; Wang et al., in [5], focus their studies in a Non-Singleton T3FLSs applied in the Industry; Alattas et al., in [6], study an implementation of a T3 FLS for MEMSs Gyroscopes; Cao et al., in [7], study a T3FLS to applied to modeling/prediction; Tian et al., in [8], propose a T3FLS in the implementation of Modeling Problems; Mohammadzadeh et al., in [9], analyze an IT3FLS and their study case; and Ma et al., in [10], implemented an Optimal T3FlS for solving singular multi-pantograph equations. Other important works based on controlling the uncertainty applied to various problems related to soft computing and fuzzy control are presented in [11,12,13,14,15,16].

This new methodology, called IT3FLS, has been used and implemented in the stabilization of various problems in the field of control; a T3 FLS applied in hybrid systems is presented in [17], an IT3FC is presented in [18], an IT3FC to improve of Image Quality is proposed in [19], a T3FS Machine Learning is implemented in [20], an IT3 fuzzy control system is studied in [21], a T3FC for Nonlinear Systems is implemented in [22], a T3FC for Time-Delay Multi-Agent Systems is analyzed in [23], a T3FC for Multi-Agent Systems is proposed in [24] and an innovator Model Predictive T3FC is implemented in [25].

One of the most studied control problems is the stabilization of an AMR; this problem in the field of the control allows to analyze the trajectory and can present different factors within the development environment, such as obstacles, objects and sensors. These factors are implemented as perturbations and are simulated through the uncertainty that the fuzzy sets allow to control and to stable more efficiently. Some important works: Tian et al. stabilize autonomous vehicles implemented an Interval Type-3 Fuzzy in [26], Castillo et al. implement an IT2FLS for AMR navigation in [27], Wang et al. proposed an FCS to help in the visualization of the navigation of AMR based on Kalman filter in [28], Pattnaik et al. study a multi-objective approach to improve the path planning of AMR in [29], Nguyen et al. implement an AMR Navigation in [30] and Joon et al. design of AMR to improve the navigation with obstacles in [31].

The meta heuristic algorithm can be an efficient methodology in the field of soft computing to solve complex problem. The BCO is one algorithm that has demonstrated excellent results in different problems, such as: in [32] where this algorithm is applied in the traffic control proposed by Jovanović et al. in 2022, in [33] the BCO is applied in the research of motion behavior through a strategy proposed by Chen in 2022, in [34] a hybridization of the BCO and T2F is implemented for measuring the efficiency of the speed in a vehicle proposed by Čubranić-Dobrodolac et al., in 2022, and in [35] a GT2FL approach for parameter adaptation in BCO utilized for FC design is proposed by Castillo et al. in 2018. This algorithm presents a good performance and convergence with excellent results.

An excellent contribution in recent years is the innovation of the dynamic adjustment in the principal parameters that had an influence in the exploration and exploitation in the performance of the algorithms. This idea is frequently implemented with fuzzy sets, which allow better management of uncertainty in problems, some important works are mentioned; in [36], Fuzzy Dynamic Parameter Setting applied to a Metaheuristic for Fuzzy Tracking Control is presented; in [37], a shadowed T2FS for dynamic parameter adaptation in two metaheuristics algorithms for optimal design of FC is offered; in [38], a comparison of T2FSs with three bio-inspired techniques in design of FCs is outlined; in [39], FSs in parameter adaptation of a BCO for controlling the trajectory of an AMR are presented; and in [40], a T3FLS optimized by a correntropy-based Kalman filter is offered.

Motivated by the excellent results obtained in [35], the first idea and contribution in this paper is the implementation of an IT3FLS for finding the optimal alpha (α) and beta (β) parameter values in BCO, during execution, which allow stabilization of the trajectory in controlling an AMR. In addition, some perturbations were added to test the Fuzzy Logic Controller (FLCS) and prove the efficiency that the IT3FLS offers in the presence of uncertainty. A comparison with other types of Fuzzy Sets, such as T1FLS, IT2FLS and GT2FLS and with the original BCO in optimizing the control of the robot, is presented, showing the potential of type-3 in this problem.

The remainder of the article is arranged as follows. Section 2 outlines Fuzzy Sets terminology, Section 3 summarizes the control case to be optimized, Section 4 outlines the proposed Fuzzy BCO algorithm, Section 5 shows the experimental results found by Fuzzy BCO with IT3FLS, Section 6 summarizes statistical tests and Section 7 mentions some conclusions.

## 2. Fuzzy Sets

Here the evolution of fuzzy sets is presented.

### 2.1. Type-1 Fuzzy Logic System

The idea of T1FLS was created by Lofti Zadeh in 1965 [41,42,43,44]. A T1FLS is defined by the universe X with a MF $\mu_{A}\left(x\right)$ with an interval of [0,1] in the values and is presented by Equation (1).

(1) $$A=\{(x,\mu_{A}\left(x\right))\;|\;x\;\in \;X\}$$

where $\mu_{A}:X\to \left[0,1\right]$.

**Figure 1:**
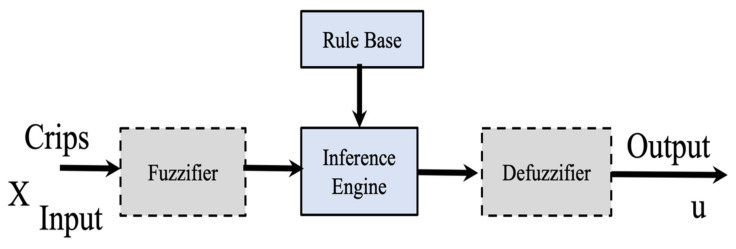
Structure of a T1FLS.

In this expression $\mu_{A}\left(x\right)$ indicates the Membership Degree (MD) of x ∈X to the set A. The expression implemented is the following: $A\left(x\right)=\mu_{A}\left(x\right)$ for all x ∈X. Figure 1 illustrated the T1FLS.

### 2.2. Interval Type–2 System

**Figure 2:**
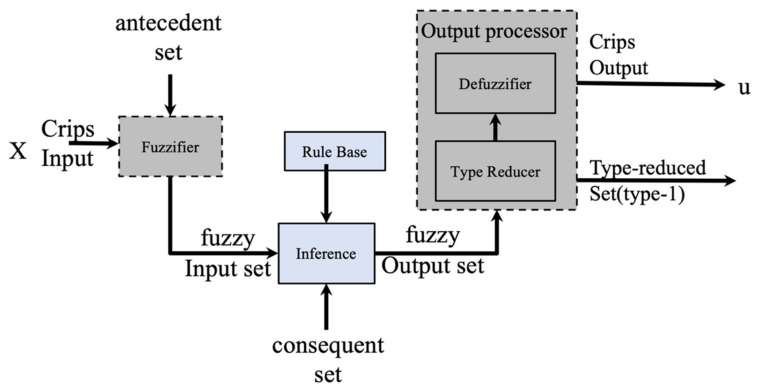
Structure of an IT2FLS.

Mendel et al., studied the main ideas of Zadeh to build and create the mathematical expression of a T2 FS, as follows [45,46]. An IT2FS $\tilde{A}$, expressed by ${\underline{\mu }}_{\tilde{\;A}}{\left(x\right)}_{\;}$and ${\underline{\mu }}_{\tilde{A}}{\left(x\right)}_{\;}$is expressed by the lower and upper MFs of $\mu_{\tilde{A}}\left(x\right)$. Where x ∈X. Equation (2) expressed the mathematical representation of an IT2FS [47].

(2) $$\tilde{A}=\left\{\left(\left(x,u\right),1\right)|\;\forall x\in X,\;\forall u\in \;J_{x}\;\subseteq \;\left[0,1\right]\right\}$$

where the primary and the secondary domain are X and Jx, respectively. All secondary degrees $\left(\mu_{\tilde{A}}\left(x,u\right)\right)$ are equal to 1. Figure 2 illustrates the overall idea of an IT2FLS.

Based on Figure 2 the output processor contains two blocks that generates a T1FS [47,48,49]. An IT2FLS is also defined by IF-THEN rules.

### 2.3. Generalized Type–2 System

**Figure 3:**
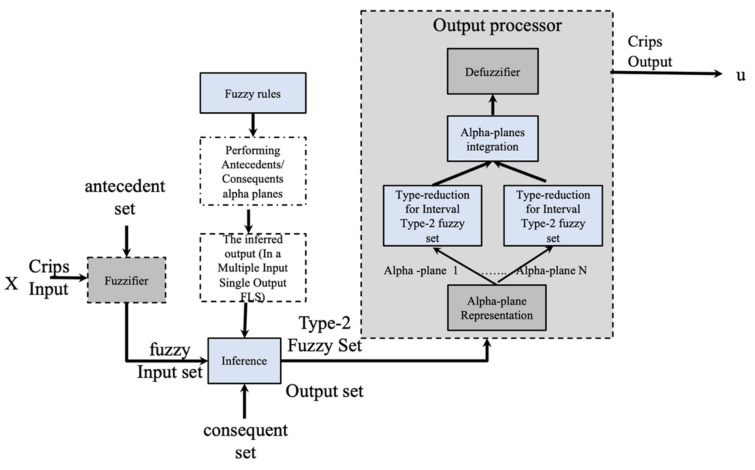
Structure of a GT2FLS.

GT2FLS presents a similarity to the T1FLS and IT2FLS, but this FLS allows evaluation of uncertainty levels with better precision, thus, their operations are different [48,49]. GT2FSs are expressed by Equation (3):

(3) $$\tilde{\tilde{A}}=\left\{\left(\left(x,u\right),\mu_{\tilde{A}}\left(x,u\right)\right)|\;\forall x\in X,\;\forall u\in \;J_{x}\;\subseteq \;\left[0,1\right]\right\}$$

where $J_{x}\subseteq \left[0,1\right]$, the partition and secondary MF are represented by *x* and *u*, which is related to the third dimension. GT2FS uses $f_{x}\left(u\right)$, in the vertical axis. Figure 3 illustrates the overall idea of a GT2FLS.

#### 𝛼-Planes Representation

**Figure 4:**
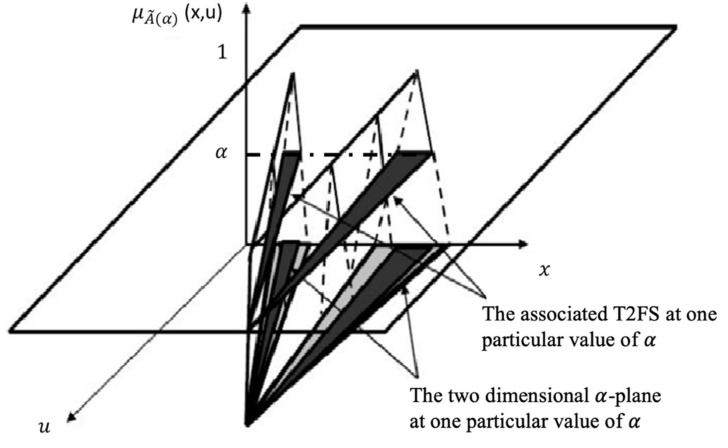
A representation of the associated T2 FS for the α-plane.

In this case, the notation for α-plane for a GT2 FLS is Ãα, and it is the union of all primary MFs of Ã, which secondary MDs are equal or higher to α (0 ≤ α ≤ 1) [50,51]. The representation of an α-plane is expressed by Equation (4) and Figure 4 [52,53].

(4) $${Ã}_{\alpha }=\left\{\left(x,u\right),\mu_{Ã}\left(x,u\right)\geq \alpha |\forall x\in X,\forall u\in J_{X\;}\subseteq \left[0,1\right]\right\}$$

### 2.4. Interval Type–3 System

**Figure 5:**
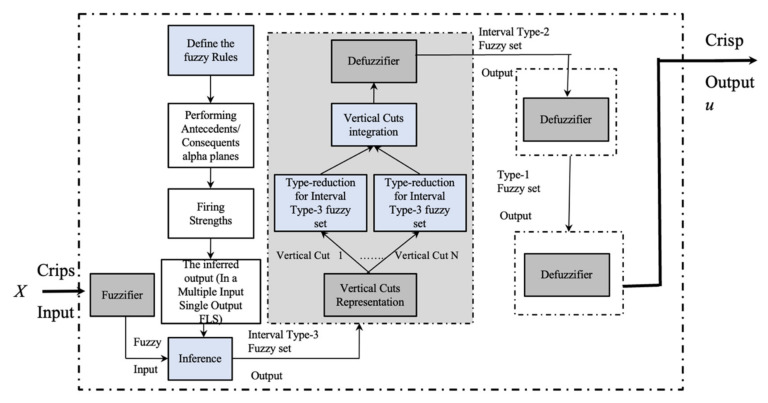
Structure of an IT3FLS.

In the last two years, an IT3 FS has proven to be very accurate in the analysis and control of uncertainty. Several research works have been focused on using this type of fuzzy sets [1,2,3,4,5,8,10,52]. Figure 5 illustrates a general structure of an IT3FLS.

#### 2.4.1. Fuzzification

**Figure 6:**
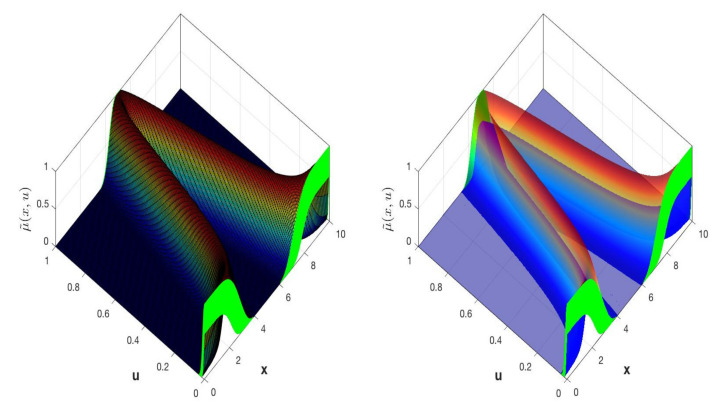
Isosurface of the MF of the IT3 FS.

An IT3 FS, represented by A, is an isosurface with a function, called MF of A. Figure 6 shows the representation, over the Cartesian product $X\times \left[0,1\right]$ in [0,1], where the universe X in the primary variable is expressed by x. The MF of A is expressed by ${\tilde{\mu }}_{A}\left(x,u\right)$, (or $\mu_{\tilde{A}}$ for simplicity) and it is called IT3 MF [52]. In other words, Equation (5) expresses the representation:

(5) $$A=\{\left(\left(x,u\right),{\;\tilde{µ}}_{A}\left(x,u\right)\right)|x\in X,\;u\in U\equiv \left[0,1\right]\}$$

in which$\;{\tilde{\mu }}_{A}\left(x,u\right)\subseteq \left[0,1\right]$. *U* is the universe for the secondary variable *u*, and the variable *U* is [0,1] is assumed in this paper. An IT3 FS, A is formulated by Equations (6) and (7).

(6) $$A=\int_{x\in X}\int_{u\in \left[0,1\right]}{\tilde{\mu }}_{A}\left(x,u\right)/\left(x,u\right)=\int_{x\in X}\mu_{A\left(x\right)}\left(u\right)/x=\int_{x\in X}\left[\int_{u\in \left[0,1\right]}{\tilde{f}}_{x}\left(u\right)/u\right]/x$$

where $\mu_{A\left(x\right)}\left(u\right)$ is an IT2 FS.

(7) $$\mu_{A\left(x\right)}\left(u\right)=\int_{u\in \left[0,1\right]}{\tilde{f}}_{x}\left(u\right)/u=\int_{u\in \left[{\underline{f}}_{x}\left(u\right),{\bar{f}}_{x}\left(u\right)\right]}1/u$$

and ∬ indicates the union over all the admissible *x* and *u*.

**Figure 7:**
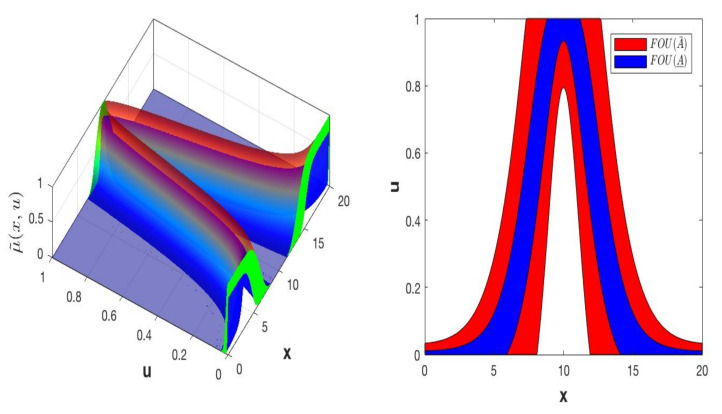
The isosurface of the left plot shows the IT3 MF of an IT3 FS and the right plot shows the $FOU\left(A\right)$, where $FOU\left(\underline{A}\right)\subseteq FOU\left(\bar{A}\right)$.

The 3D plot of the IT3MF is an isosurface formed by all the secondary IT2MFs $\mu_{A\left(x\right)}\left(u\right)$ indicated in green color in Figure 7.

In this paper, an IT3 singleton fuzzifier is used, which has a single point of nonzero membership [52]. This paper is based on an extension and improvement of the work in [35], as the methodology is similar. In [35], a non-singleton GT2FLS was used to simplify computations. Now, in the proposed method in this work, a comparison of results obtained in [35] and the approach that a singleton IT3FLS is presented. Based on experimental results, one important characteristic that a singleton IT3FLS provides is the ability to simplify the computing processes and improve efficiency, as it assumes the utilization of numeric precise values in the input data.

#### 2.4.2. Inference

When the variables (inputs and outputs) have been created, with each MFs, the second process is called the inference. The structure of the rules in the IT3FLS is the standard Mamdani-type FLS rules are used in the T1FLS, IT2FLS and GT2FLS. The antecedents and the consequents sets are expressed by an IT3 FS. Therefore, for a type-3 FLS with n inputs $x_{1}\;\epsilon \;X_{1},\;\ldots ,\;x_{n}\;\in \;X_{n\;}$and one output y∈Y, Multiple Input Single Output (MISO), if we assume there are M rules, the kth rule in the IT3FLS can be formulated with Equation (8) [54]:

(8) $$R_{z}^{k}:IF\;x_{1}\;is\;F_{i}^{k}and\ldots and\;x_{i}\;is\;F_{i}^{k}\;and\ldots and\;x_{n}\;is\;F_{n}^{k}\;THEN\;y_{1}\;is\;G_{1}^{k},\ldots ,\;y_{j}is\;G_{j}^{k},\ldots ,y_{m}is\;G_{m}^{k}$$

where i = 1, …, n (inputs), j = 1, …, m (outputs) and k = 1, …, r (rules).

The trigger force ${\tilde{\Phi }}^{k}\left(x^{\prime }\right)$, is the value of IT2 MF of the operation ⨅ of all the membership values of the antecedents $\mu_{F_{i}^{k}}\left(x_{i}\right)$ that contributes to the activation level of the rule described by Equation (9).

(9) $${\tilde{\Phi }}^{k}\left(x^{\prime }\right)={⨅}_{i=1\;\;}^{n}\mu_{F_{i}^{k}}\left(x_{i}\right)\;$$

The trigger level of the rule is the membership value $\mu_{B_{j}^{k}}(y_{i}|x^{\prime })$ resulting from the operation ${\tilde{\Phi }}^{k}\left(x^{\prime }\right)$ and the value of the membership of the consequent of the rule $\mu_{G_{j}^{k}}\left(y_{j}\right)$. That is, the composition operation (∘) between the facts and the rules of the knowledge base that describes the relation $B_{j}^{k}=A_{x^{\prime }}\circ \;{\mathbb{R}}_{j}^{k}$, where $A_{x^{\prime }}$ is a singleton fuzzy, Equation (10) shows this representation.

(10) $$\mu_{B_{j}^{k}}\left(y_{i}|x^{\prime }\right)={\tilde{\Phi }}^{k}\left(x^{\prime }\right)⊓\mu_{G_{j}^{k}}\left(y_{j}\right)$$

The method to combine the rules is using the join operation (⊔) “fuzzy union” with the agregation operation which allows calculation of the aggregation of the values of $\mu_{B_{j}^{k}}\left(y_{i}|x^{\prime }\right)$, are expressed by Equations (11)–(14).

(11) $$B_{j}=B_{j}^{1}⋃\ldots ⋃B_{j}^{k}⋃\ldots ⋃B_{j}^{r}={⋃}_{k=1}^{r}B_{j}^{k}$$

(12) $$\mu_{B_{j}}\left(y_{i}|x^{\prime }\right)=\mu_{B_{j}^{1}}\left(y_{j}|x^{\prime }\right)⊔\ldots ⊔\mu_{B_{j}^{k}}\left(y_{j}|x^{\prime }\right)⊔\ldots ⊔\mu_{B_{j}^{r}}(y_{j}|x^{\prime })$$

(13) $$\mu_{B_{j}}\left(y_{j}|x^{\prime }\right)={⊔}_{k=1}^{r}\mu_{B_{j}^{k}}(y_{j}|x^{\prime })$$

(14) $$\mu_{B_{j}}\left(y_{j}|x^{\prime }\right)={⊔}_{k=1}^{r}\left[{\tilde{\Phi }}^{k}\left(x^{\prime }\right)⊓\mu_{G_{j}^{k}}\left(y_{j}\right)\right]$$

#### 2.4.3. Vertical Slice Representation

The mathematically representation for a T3 FS used in this paper is the union of vertical slices, where each slice is an embedded T2 FS, and this method is represented for an IT3FS [53,54,55].

**Figure 8:**
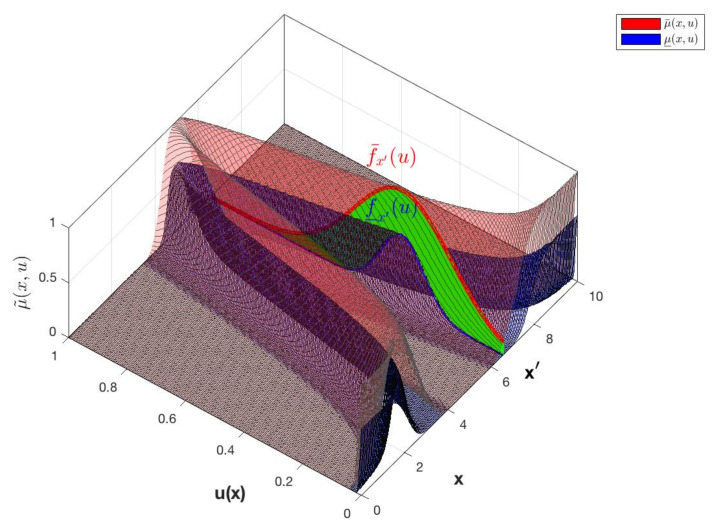
IT3 FS, A, with IT3 MF, ${\tilde{\mu }}_{A}\left(x,u\right)$ and an embedded vertical cut, $\mu_{A\left(x^{\prime }\right)}\left(u\right)\in \left[{\underline{f}}_{x^{\prime }}\left(u\right),\;{\bar{f}}_{x^{\prime }}\left(u\right)\right]$ with the FOU in green color.

**Figure 9:**
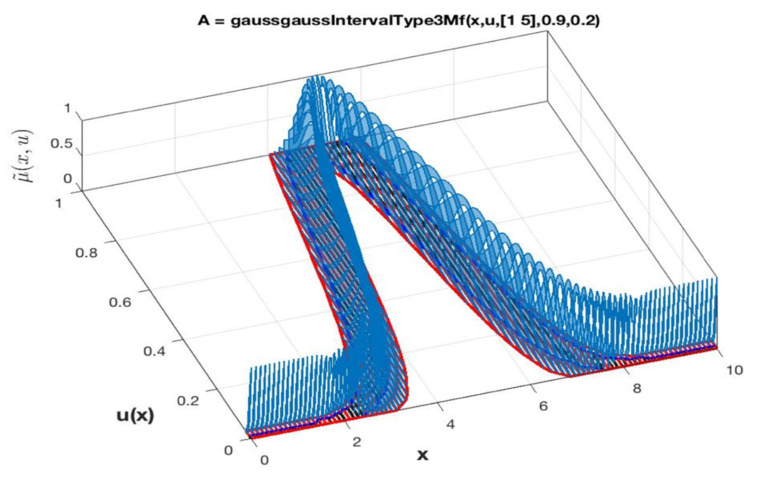
IT3 FS, A, illustrated by the vertical cuts IT2 FSs with embedded MFs, ${\tilde{f}}_{x}\left(u\right)$.

Using the above methods, an IT3 FS, A, is represented by the union of vertical slices [56], of the lower T2MF, ${\underline{\mu }}_{A}\left(x,u\right)$ and upper, ${\bar{\mu }}_{A}\left(x,u\right)$, where each vertical slice is a Upper and Lower T1 FS (Figure 8). Moreover, by the union of vertical slices of, 𝔸, where each vertical slice is an IT2 FS (Figure 9).

The vertical slice based on each value of the primary variable x for an IT3FLS and the union of its secondary IT2FLS is expressed in Figure 9. Vertical slice is represented by Equation (15).

(15) $$A=\int_{x\in X}\mu_{A\left(x\right)}\left(u\right)/x$$

where

$$\mu_{A\left(x\right)}\left(u\right)=\int_{x\in J_{x}}{\tilde{f}}_{x}\left(u\right)/u=\int_{u\in \left[{\underline{f}}_{x}\left(u\right),{\bar{f}}_{x}\left(u\right)\right]}1/u$$

#### 2.4.4. Type Reductor

The block on the type reduction is performed based on the Karnik and Mendel algorithm [55,56,57], and expressed by Equations (16)–(19).

(16) $$\underline{y_{j}^{l}}=\frac{\sum_{k=1}^{n_{level}}\alpha_{k}{\underline{y}}_{{\underline{B}}_{j}}^{\alpha_{k}}}{\sum_{k=1}^{n_{level}}\alpha_{k}}$$

(17) $${\underline{y}}_{j}^{r}=\frac{\sum_{k=1}^{n_{level}}\alpha_{k}{\bar{y}}_{{\underline{B}}_{j}}^{\alpha }}{\sum_{k=1}\alpha_{k}}$$

(18) $${\bar{y}}_{j}^{l}=\frac{\sum_{k=1}^{n_{level}}\alpha_{k}{\underline{y}}_{B}^{\underline{\alpha }}}{\sum_{k=1}^{n_{level}}\alpha_{k}}$$

(19) $${\bar{y}}_{j}^{r}=\frac{\sum_{k=1}^{n_{level}}\alpha_{k}{\bar{y}}_{B}^{\underline{\alpha }}}{\sum_{k=1}^{n_{level}}\alpha_{k}}$$

#### 2.4.5. Defuzzification

The last process is aimed at calculating the average of $y_{l}$ and $y_{r}$, to determine the defuzzified output of an IT3FS singleton FLS [52]. Equation (20) shows the mathematical representation:

(20) $$\;{\hat{y}}_{j}=\frac{\left({\underline{y}}_{j}^{l}+{\underline{y}}_{j}^{r}\right)+\left({\bar{y}}_{j}^{l}+{\bar{y}}_{j}^{r}\right)}{4}$$

### 2.5. Mathematical Representation for ScaleTriScaleGaussIT3MF

**Figure 10:**
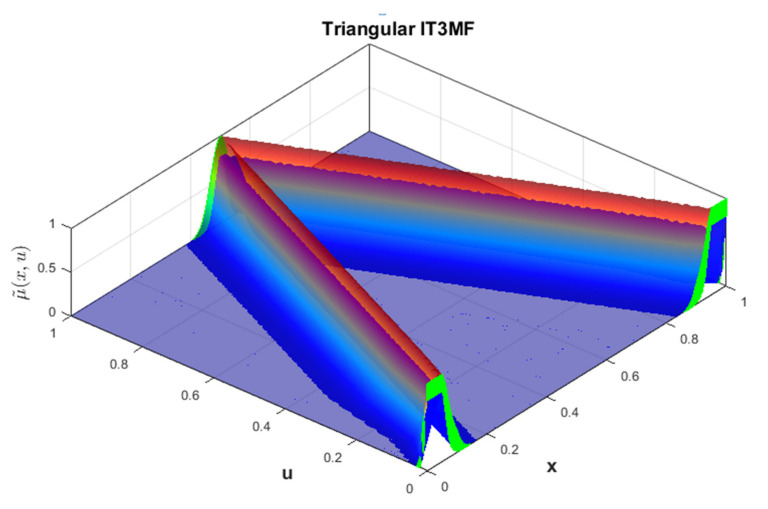
Visual representation of the ScaleTriScaleGaussIT3MF IT3FS.

${\tilde{\mu }}_{A}\left(x,u\right)=$ ScaleTriScaleGaussIT3MF (x,{{[a_1_, b_1_, c_1_]}, λ,$\;\left[l_{1},l_{2}\right]$}) is the presentation of the Interval Type-3 Triangular MF denoted by ${\tilde{\mu }}_{A}\left(x,u\right)$ = ScaleTriScaleGaussIT3MF designed by the triangular FOU (A) and has the parameters $\left[a_{1},b_{1},c_{1}\right]$ (UpperParameters) for the UMF, and the parameters are λ (LowerScale) and l (LowerLag) for LMF, which design the $DOU=\left[\underline{\mu }\left(x\right),\bar{\mu }\left(x\right)\right]$. The vertical cuts $A_{\left(x\right)}\left(u\right)$ are defined by $FOU\left(A\right)$, which is IT2FS with gaussian IT2 MF, $\mu_{A\left(x\right)}\left(u\right)$ with parameters $\left[\sigma_{u},m\left(x\right)\right]$ for the UMF and λ (LowerScale) and l (LowerLag) for LMF. The IT3MF ${\tilde{\mu }}_{A}\left(x,u\right)=$ ScaleTriScaleGaussIT3MF (x,{{[a_1_, b_1_, c_1_]}, λ,$\;\left[l_{1},l_{2}\right]$}); Figure 10 illustrates the visual representation and the Equation (21) shows more detail in the description of this MF.

(21) $$\bar{\mu }\left(x\right)=\left\{\begin{array}{cc} 0 & x<a_{1}\\ \frac{x-a_{1}}{b_{1}-a_{1}} & a_{1}\leq x\leq b_{1}\\ \frac{c_{1}-x}{c_{1}-b_{1}} & b_{1}<x\leq c_{1}\\ 0 & x>c_{1} \end{array}\right.$$

The LMF of DOU, $\underline{\mu }\left(x\right)$ is defined by the $a_{2}$ and $c_{2}$ values, design by the $\left(a_{1},b_{1},c_{1}\right)$ parameters to UMF of the DOU, $\bar{\mu }\left(x\right)$, and the elements of the vector lowerLag $\left(l\right)$. The Equation (22) shows the representation:

(22) $$a_{2}=b_{1}-\left(b_{1}-a_{1}\right)\left(1-l_{1}\right)\;c_{2}=b_{1}+\left(c_{1}-b_{1}\right)\left(1-l_{2}\right)\;\mu \left(x\right)=\left\{\begin{array}{cc} 0 & x<a_{2}\\ \frac{x-a_{2}}{b_{1}-a_{2}} & a_{2}\leq x\leq b_{1}\\ \frac{c_{2}-x}{c_{2}-b_{1}} & b_{1}<x\leq c_{2}\\ 0 & x>c_{2} \end{array}\right.$$

Function $\mu \left(x\right)$ is multiplied by the λ parameter to design the LMF of the DOU, $\underline{\mu }\left(x\right)$, represented by: $\underline{\mu }\left(x\right)=\lambda \;\mu \left(x\right)$. Thus, $\bar{u}\left(x\right)$ y $\underline{u}\left(x\right)$ are the Lower and Upper limits of the DOU. The range $\delta \left(u\right)$ and $\sigma_{u}$ radius of the FOU are expressed in Equations (23) and (24):

(23) $$\delta \left(u\right)=\bar{u}\left(x\right)-\underline{u}\left(x\right)$$

(24) $$\sigma_{u}=\frac{\delta \left(u\right)}{2\sqrt{3}}+\varepsilon$$

where ε is an epsilon of the computer.

The apex or nucleus of $m\left(x\right)$ in the IT3 MF $\tilde{\mu }\left(x,u\right)$, is expressed by the Equation (25):

(25) $$m\left(x\right)=\left\{\begin{array}{cc} 0 & x<a\\ \frac{x-a}{b_{1}-a} & a\leq x\leq b_{1}\\ \frac{c-x}{c-b_{1}} & b_{1}<x\leq c\\ 0 & x>c \end{array}\right.$$

where $a=\left(a_{1}+a_{2}\right)/2$ and $c=\left(c_{1}+c_{2}\right)/2$. Therefore, the vertical cuts with IT2 MF, $\mu_{A\left(x\right)}\left(u\right)=\left[\;{\underline{\mu }}_{A\left(x\right)}\left(u\right),{\bar{\mu }}_{A\left(x\right)}\left(u\right)\right]$ are described by Equations (26) and (27):

(26) $${\bar{\mu }}_{A\left(x\right)}\left(u\right)=exp\left[-\frac{1}{2}{\left(\frac{u-m\left(x\right)}{\sigma_{u}}\right)}^{2}\right]$$

(27) $${\underline{\mu }}_{A\left(x\right)}\left(u\right)=\lambda \cdot exp\left[-\frac{1}{2}{\left(\frac{x-m\left(x\right)}{\sigma_{u}^{*}}\right)}^{2}\right]$$

where $\sigma_{u}^{*}=\sigma_{u}\;\sqrt{\frac{\ln\left(l\right)}{\ln\left(\varepsilon \right)}}$, $l=\left(l_{1}+l_{2}\right)/2$. If l=0 then $\sigma_{u}^{*}=\sigma_{u}$. Therefore ${\bar{\mu }}_{A\left(x\right)}\left(u\right)$ and ${\underline{\mu }}_{A\left(x\right)}\left(u\right)$ are the UMF and LMF of the IT2FS with the vertical cuts of the secondary IT2MFs of the IT3FS.

**Figure 11:**
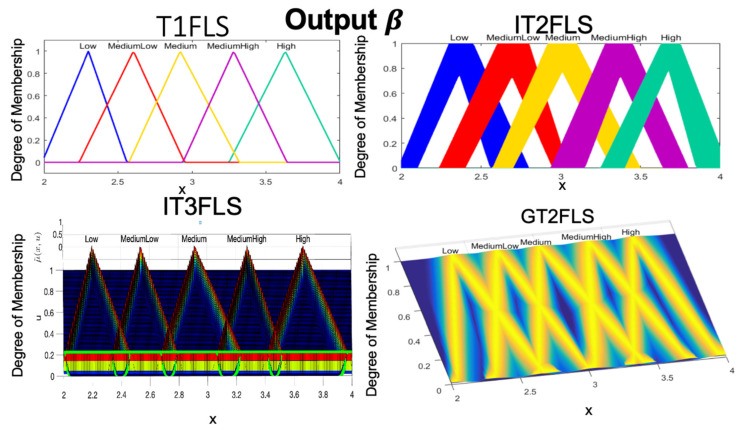
Visual representation of the Triangular MF (output 1-β) for each Fuzzy Sets.

A visual representation of triangular FMs for each fuzzy sets is illustrated in Figure 11.

## 3. Study Case

### 3.1. Fuzzy Controller

**Figure 12:**
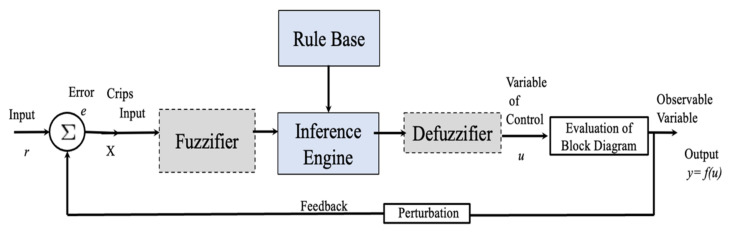
T1FLS applied in a FLC.

A field in soft computing is the control of a non-linear plant to stabilize real systems, such as airplane simulators, arms robot and trajectories of robots. Ebrahim Mamdani in 1974 created the area of FLC to control plants [58,59,60,61,62]. Figure 12 represents a T1FLC.

At present, a real control problem is analyzed and implemented in this paper with the goal to evaluate the performance of the IT3FLS with the hybridization of BCO, the general description is outlined in the following section.

### 3.2. Mobile Robot Controller

**Figure 13:**
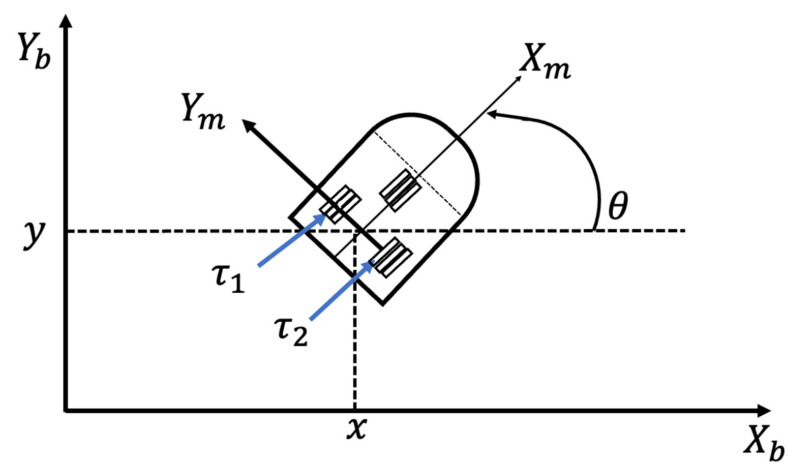
Graphical idea for the study problem.

A unicycle mobile robot is the studied problem [35], which consists of two driving wheels with the same axis and one front free wheel, and Figure 13 illustrates a representation of this robot.

In this case, the robot model can assume this motion, as shown by Equations (28) and (29).

(28) $$M\left(q\right)\dot{v}+C\left(q,\dot{q}\right)v+Dv=\tau +P\left(t\right)$$

where, $q={\left(x,y,\theta \right)}^{T}\;$is the vector of coordinates, $\upsilon ={\left(v,w\right)}^{T}$is the vector of velocities, $\tau =\left(\tau_{1},\tau_{2}\right)\;$is the vector of torques applied to the wheels, where $\tau_{1}$ and $\tau_{2}$ indicates the torques of the left and right wheels, $P\in R^{2}\;$is the uniformly bounded disturbance vector, $M\left(q\right)\in R^{2\times 2}$ is the positive-definite inertia matrix, $C{\left(q,\dot{q}\right)}^{\vartheta }$ is the vector of centripetal and Coriolis forces and $D\in R^{2\times 2}$ is a diagonal positive-definite damping matrix. Equation (29) expresses the kinematic system.

(29) $$\dot{q}=\underset{J_{q}}{\underbrace{\left[\begin{array}{c} \cos\theta \;0\\ \sin\theta \;0\\ 0\;1 \end{array}\right]}}\underset{\nu }{\underbrace{\left[\begin{array}{c} v\\ w \end{array}\right]}}$$

where, $\left(x,y\right)$ is the position in the X–Y (world) reference frame, θ is the angle between the heading direction and the x-axis, ν and w are the linear and angular velocities.

Furthermore, Equation (30) expresses the non-holonomic constraint, which corresponds to a no-slip wheel condition.

(30) $$\dot{y\;}\cos\theta -\dot{x}\;\sin\;\theta =0$$

**Figure 14:**
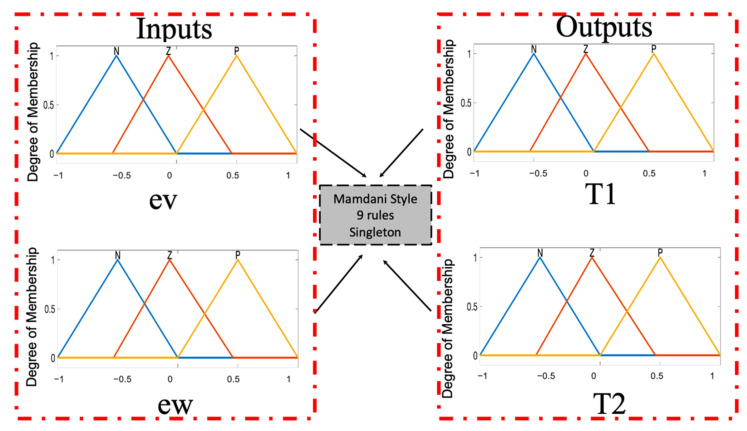
General idea of the Case Study.

**Figure 15:**
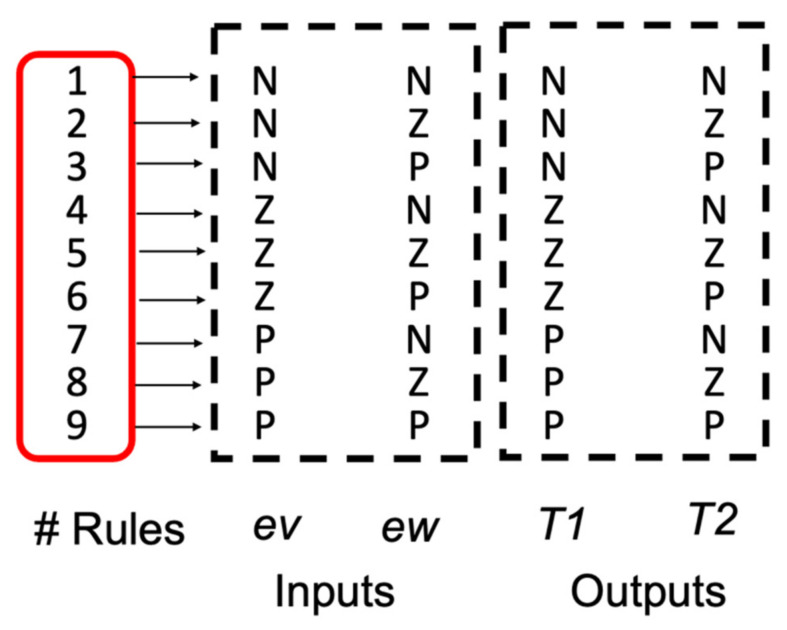
Fuzzy rules of the FC for the AMR Controller.

The T1FLS contains two inputs: ev (error in the linear velocity) with three MFs called N(Negative), Z(Zero) and P(Positive), and ew (error in the angular velocity) with three MFs with the same values. The outputs are: T1 (Torque 1), and T2 (Torque 2), which are designed with three triangular MFs called N, Z, P. The T1FLS and the Fuzzy Rules are illustrated in Figure 14 and Figure 15 and their description can be found in [36,37].

**Figure 16:**
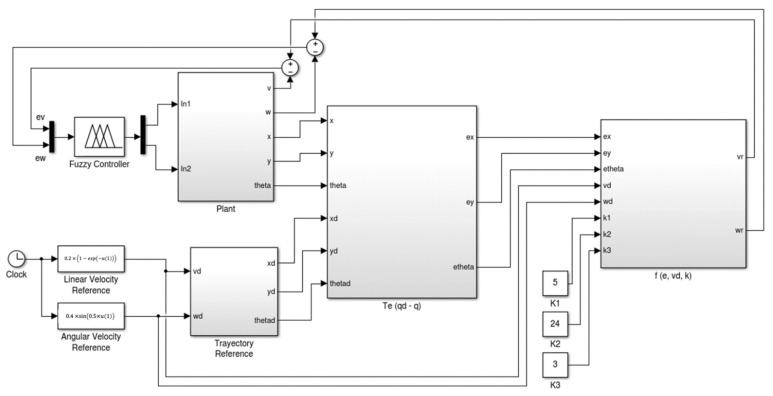
Representation in the model for the control problem.

**Figure 17:**
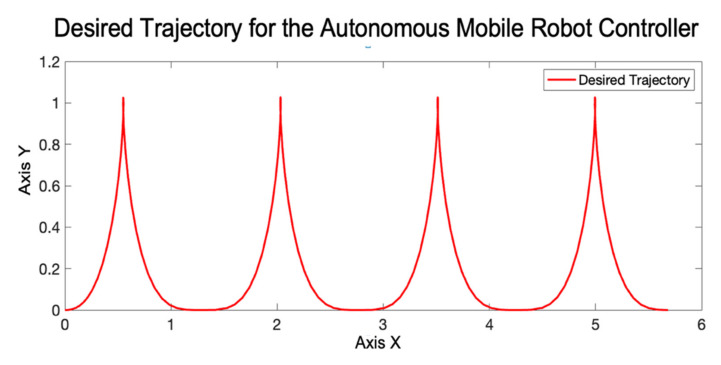
Reference trajectory for the AMR.

With the fuzzy rules shown in Figure 15, the evaluation of the IT3FLS is simulated in the representation that is illustrated the model for this control problem by Figure 16, and the goal of the AMR consists in following a trajectory based on a reference. Figure 17 shows in red color the reference in the desired trajectory of the AMR.

## 4. Fuzzy BCO

The BCO algorithm was created by Teodorović in 2009, and its main function is to mimic the behavior that the bees have in finding the food [63]. Honeybees use a variety of methodologies, such as the waggle dance, with the goal being the localization of food source. This algorithm has some characteristics, such as: being robust, simple and population based stochastic [64].

### 4.1. Original BCO Algorithm

**Figure 18:**
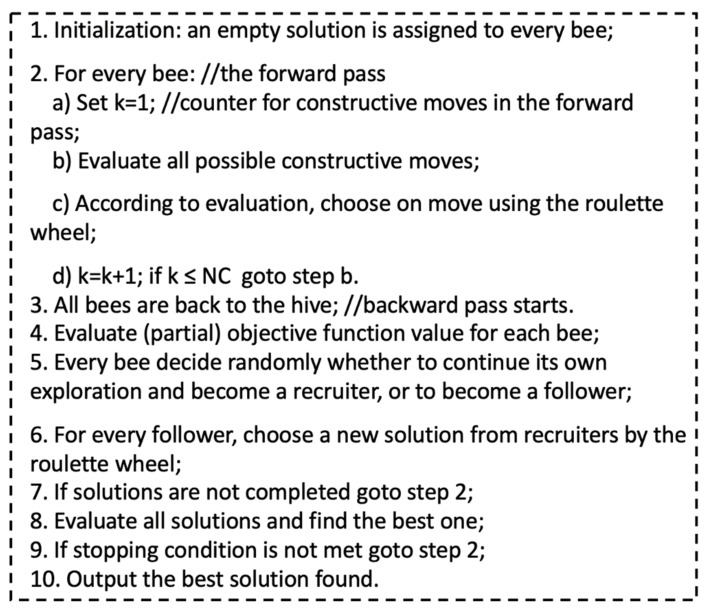
Sequential steps of the BCO Algorithm.

The principles that form the BCO algorithm are based on creating a colony of artificial bees (multi agent system) with the ability to solve different types of problems [64,65]. In BCO algorithm, *B* indicates the total of bees and *NC* represents the total of constructive moves occurring one forward pass. The steps are illustrated in Figure 18. The dynamics of the BCO algorithm is represented on Equations (31)–(34):

(31) $$P_{ij,n}=\frac{{\left[\rho_{ij,n}\right]}^{\alpha }\cdot \;{\left[\frac{1}{d_{ij}}\right]}^{\beta }}{\sum_{j\in A_{i,n}}{\left[\rho_{ij,n}\right]}^{\alpha }\cdot {\left[\frac{1}{d_{ij}}\right]}^{\beta }}$$

(32) $$D_{i}=K\cdot \frac{{\mathrm{Pf}}_{i}}{{\mathrm{Pf}}_{\mathrm{colony}}}$$

(33) $${\mathrm{Pf}}_{i}=\frac{1}{L_{I}},L_{i}=Tour\;Length$$

(34) $${\mathrm{Pf}}_{\mathrm{colony}}=\frac{1}{N_{\mathrm{Bee}}}\;\overset{N_{\mathrm{Bee}}}{\underset{i=1}{\sum }}{\mathrm{Pf}}_{i}$$

Equation (31) represents the probability that a bee *k* located on a node *i* selects the next node expressed by *j*, where the set of nodes connected to node *i* and relation to bee *K* is expressed by Nk_i_, and the probability to visit the next node is represented by ρ_ij_. The β is inversely proportional to the distance of the node; *d*_ij_ indicates the distance of node *i* until node *j*. In addition, α is a parameter that is utilized to identify excellent solutions. Equation (32) indicates the behavior called waggle dance that is calculated by a linear function, where K represents the scaling factor [66], Pf_i_ indicates the profitability scores of bee *i* expressed in Equation (33) and Pf_colony_ represents the bee colony’s average profitability in Equation (34) and is updated after each bee completes its tour. In this paper, the mean square error (MSE) indicates the fitness function and is used like the waggle dance for each iteration [35,39]. Thus, a bee is a vector that represents all the values of the MFs.

The structure of the T1FLS in this study case has Triangular and Trapezoidals MFs (see Figure 14), obtaining a total of 36 values.

### 4.2. Fuzzy BCO

**Figure 19:**
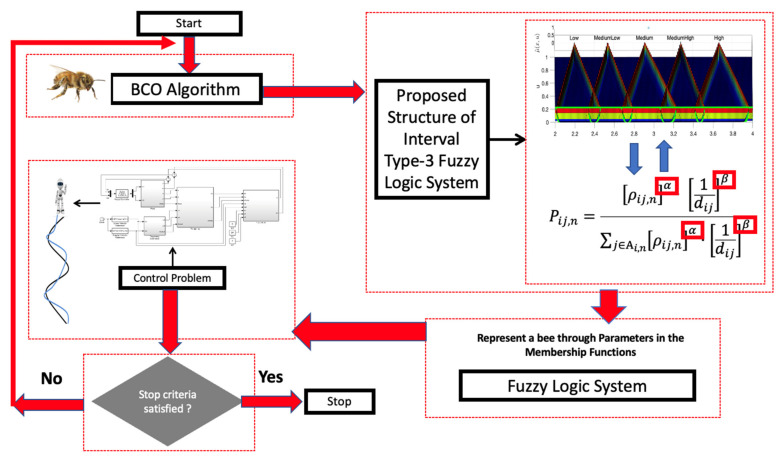
Graphical representation for the Fuzzy BCO algorithm.

The main idea is the implementation of an IT3FLS to find the optimal α and β parameter values for the error minimization in the control problem. The proposal is illustrated in Figure 19.

An input in the proposal is the Iteration, in the first executions are ‘‘low’’, and when the algorithm almost finish iterations are ‘‘high’’ or close to 100%. The idea is illustrated by Equation (35) [35]:

(35) $$\mathrm{Iteration}=\frac{Current\;Iteration}{Maximum\;of\;Iterations}\;$$

The diversity is calculated by Equation (36), this second input measures the dispersion degree of the bees. The main contribution of using diversity in the BCO algorithm is to control the possible convergence in local minimum. This behavior is verified with the design of the fuzzy rules [37].

(36) $$Diversity\left(S\left(t\right)\right)=\frac{1}{n_{s}}\;\overset{n_{x}}{\underset{i=1}{\sum }}\sqrt{X_{ij}\left(t\right)-{\bar{X}}_{j}{\left(t\right)}^{2}}$$

Based on the BCO algorithm; the current iterations is *t*, size of the population is $n_{s}$, a bee is *i*, the total of solutions is $n_{x}$, the possible next solution is *j*, $X_{ij}$ is the solution *j* of the bee *i*, and finally, $\underline{X}$_j_ the solution *j* of the best bee in the space search. The MSE is calculated by Equation (35).

(37) $$\mathrm{MSE}=\frac{1}{n}\;\overset{n}{\underset{i=1}{\sum }}{\left({\bar{Y}}_{i}-Y_{i}\right)}^{2}$$

**Figure 20:**
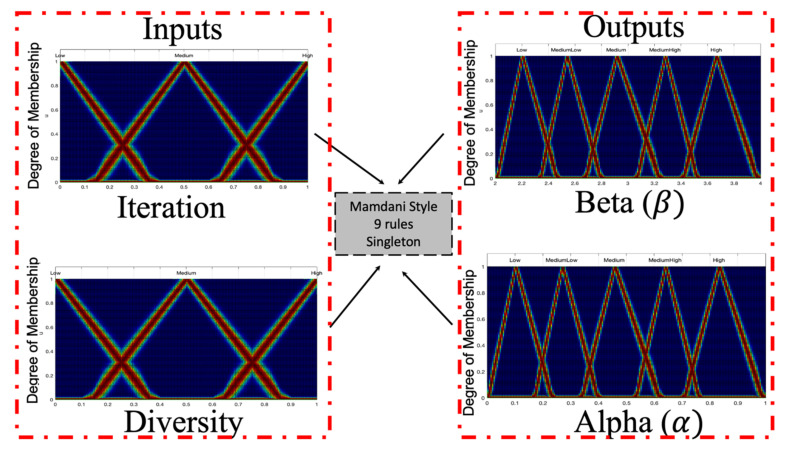
Fuzzy Proposal BCO using a IT3FLS.

**Figure 21:**
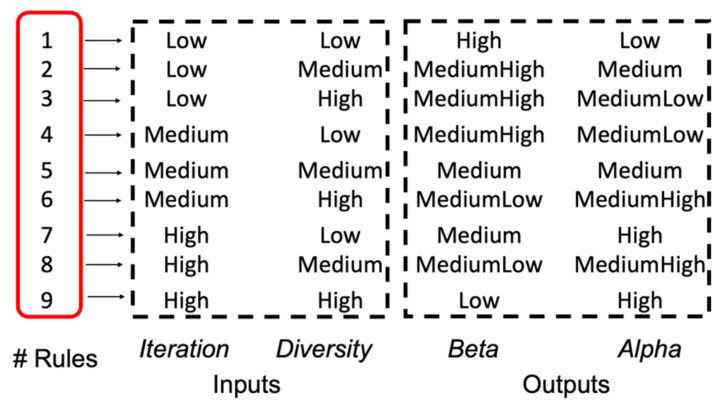
Rules for the Fuzzy BCO algorithm.

The design of the MFs is realized in a symmetrical way, and is appreciated in Figure 20 and in Figure 21 illustrates the fuzzy rules for the IT3FLS.

Several previously experiments were realized to explore the behavior in this algorithm. The main contribution to find is beginning the execution with high exploration and thus, the proposal helps to analyze all the search space. In the beginning, iteration and diversity are *Low*; this is because all bees have a random position in steps 1. This reasoning is used for realizing the Rules, based on the analysis that the *high* value for β indicates that the bee should realize high exploration and the value *low* of α indicates that the bee has small exploitation. When the Iteration is *high* (last executions), the bees present a *high* diversity, on the other hand, the value of β is *low*, then a *low* exploration and the value of α is *high* to achieve a better exploitation.

**Figure 22:**
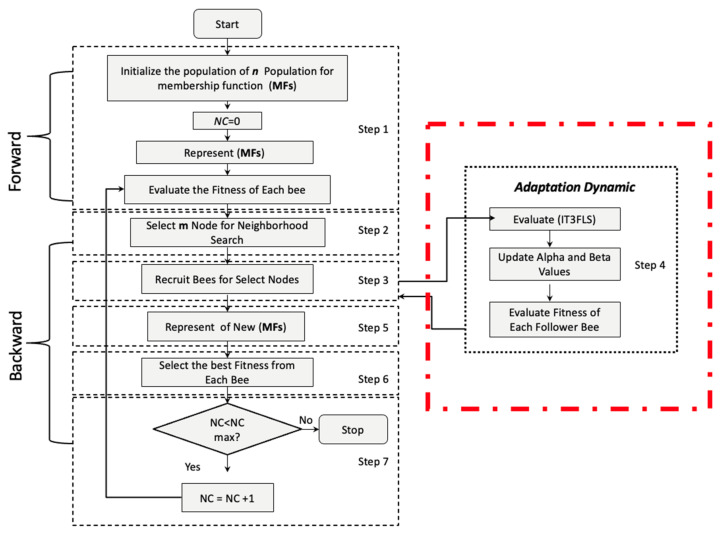
Flowchart of the Fuzzy BCO algorithm.

Based on the original BCO algorithm with the implementation of the adaptation dynamic with the IT3FLS, the flowchart of BCO is shown in Figure 22, where “*ScoutBees*” represents the population size and “*FollowerBees*” indicates the bee with a better fitness function.

## 5. Experimental Results

Experimental results were achieved with one scenario of external perturbations called pulse generated noise. Where the amplitude is set to 90, period in seconds of 100, pulse width (%) is set to 0.9 and phase delay is set to 100.

Some important metrics to evaluate the excellent performance of the FLC are: the Integral Square Error (ISE), Integral Absolute Error (IAE), Integral Time Squared Error (ITSE), Integral Time Absolute Error (ITAE) and Root Mean Square Error (RMSE) shown in Equations (38)–(42);

(38) $$\mathrm{ISE}=\overset{\infty }{\underset{0}{\int }}e^{2}\left(t\right)dt$$

(39) $$\mathrm{IAE}=\overset{\infty }{\underset{0}{\int }}{\left|e\left.\left(t\right)\right|dt\right.}_{\;}$$

(40) $$\mathrm{ITSE}=\overset{\infty }{\underset{0}{\int }}e^{2}\left(t\right)tdt_{\;}$$

(41) $$\mathrm{ITAE}=\overset{\infty }{\underset{0}{\int }}{\left|e\left.\left(t\right)\right|tdt\right.}_{\;}$$

(42) $$\varepsilon =\sqrt{\frac{1}{N}\sum_{t=1}^{N}{\left(X_{t}-{\hat{X}}_{t}\right)}^{2}}$$

The BCO algorithm was configured with the following parameters: a *population* of 50 values, size of *employed bee* of 30 values, a total of *iterations* of 30 values, and the main objective for IT3FLS to find the optimal values in α and β parameters.

The results of the experimentations are outlined in Table 1, which summarizes the average in the errors for each performance index of 30 Experiments for the AMRC controller using the Original BCO, when the α and β values are set to 0.5 and 3, respectively. Table 1 presents the average error for each fuzzy BCO; for example, FBCO-T1FLS presents the Fuzzy BCO with dynamic adjustment using the T1FLS, FBCO-IT2FLS implemented the IT2FLS, FBCO-GT2FLS using the GT2FLS and FBCO-IT3FLS implemented the proposal IT3FLS.

Table 1 shows better results when IT2FLS is simulated in the model. For example, the average in the best MSE with the Original BCO is of **2.37** **× 10^0^**, with the FBCO-T1FLS the error is **1.31** **× 10^0^**, with FBCO-IT2FLS the error is **9.83** **× 10^−1^,** with the FBCO-GT2FLS the error is **1.46** **×10^0^** and with FBCO-IT3FLS the better error is **1.19** **× 10^0^**; in these results, without perturbation in the model, the more stable results are for FBCO-T2FLS. Disturbance is added in the model to analyze the performance and the stabilization when FBCO-IT3FLS is used in the dynamic adjustment of the α and β parameters.

**Figure 23:**
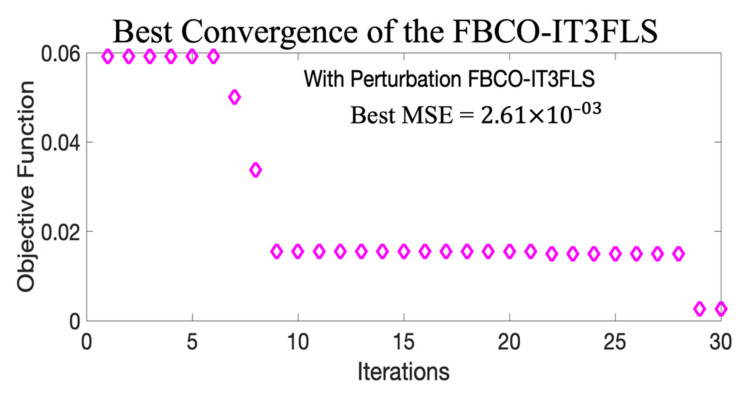
Best convergence with the proposed Fuzzy BCO with IT3FLS.

Table 2 summarizes the results when the perturbation is added and the better results are when FBCO-IT3FLS is used in the model. For example, the best Mean Square Error (MSE) with the Original BCO is **7.53** **× 10**^−3^, with the FBCO-T1FLS, FBCO-IT2FLS and FBCO-GT2FLS the errors are **1.03** **× 10^−2^**, and with FBCO-IT3FLS the best error found is **2.61** **× 10^−3^**. Figure 23 illustrates the convergence in the best result when FBCO-IT3FLS is implemented.

**Figure 24:**
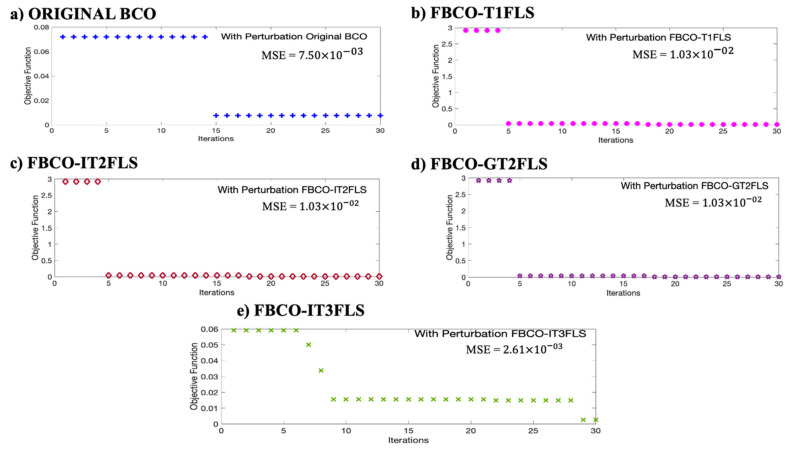
Comparative of the Best convergence for each Fuzzy BCO, (**a**) Original, (**b**) T1FLS, (**c**) IT2FLS, (**d**) GT2FLS and (**e**) IT3FLS.

Figure 23 shows a fast convergence is shown when the proposed Fuzzy BCO is executed with the IT3FLS. A comparative of the performance in each best results with perturbation in the model is shown in Figure 24.

Figure 24 shows a fast convergence when the FBCO-IT3FLS is implemented with perturbation in the model (See Figure 20e). It is interesting to observe that Original BCO presents excellent stabilization in the convergence (See Figure 20a).

**Figure 25:**
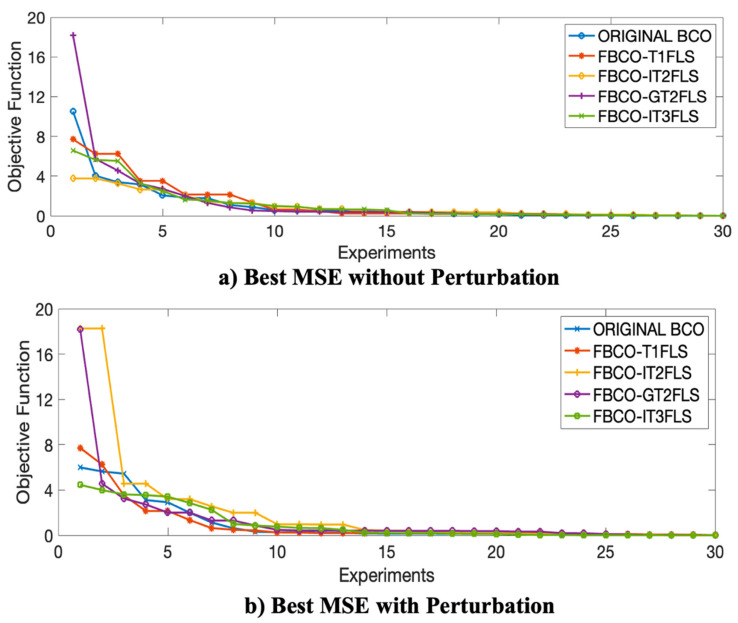
Comparative of the Best convergence for each Fuzzy Sets and original BCO algorithm, (**a**) without Perturbation and (**b**) with Perturbation.

**Figure 26:**
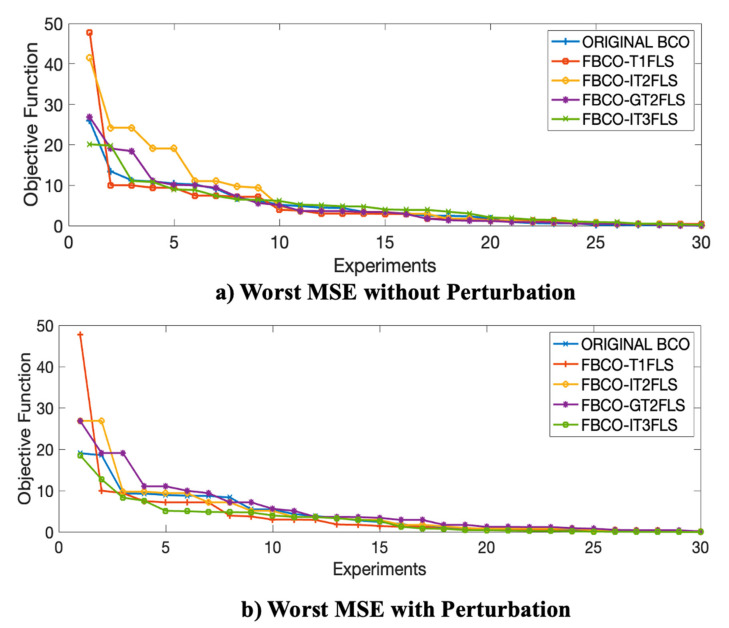
Comparative of the Worst convergence for each Fuzzy Sets and original BCO algorithm, (**a**) with Perturbation and (**b**) with Perturbation in the model.

With the goal to analyze the best and the worst results for each Fuzzy sets and comparison with Original BCO algorithm, Figure 25 and Figure 26 illustrate the best and the worst errors of the 30 experiments with and without perturbation.

In Figure 25 and Figure 26, the proposal Fuzzy BCO with IT3FLS (green line) presents the lower errors and fast convergence in the experimental results. Another important behavior of the IT3FLS is the stabilization on the errors.

**Figure 27:**
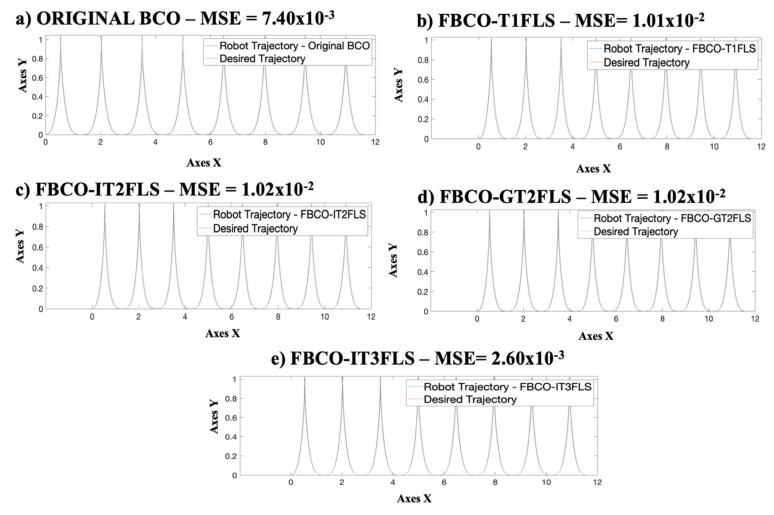
Behavior of the best trajectory for each method with perturbation. (**a**) Original BCO, (**b**) FBCO-T1FLS, (**c**) FBCO-IT2FLS, (**d**) FBCO-GT2FLS and (**e**) FBCO-IT3FLS.

An important analysis of the excellent behavior of the FBCO-IT3FLS is comparing the best trajectories in the AMR that were found for each fuzzy set and the original BCO algorithm. Figure 27 illustrates this comparative with the results when applying perturbation.

**Figure 28:**
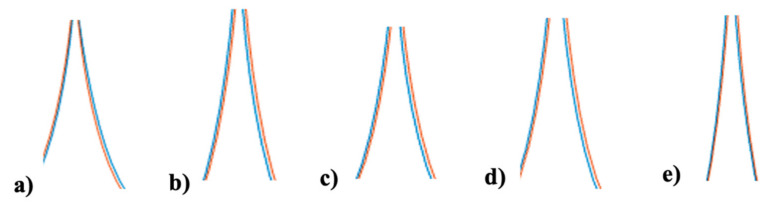
Extraction of the best trajectory for each method with perturbation. (**a**) Original BCO, (**b**) FBCO-T1FLS, (**c**) FBCO-IT2FLS, (**d**) FBCO-GT2FLS and (**e**) FBCO-IT3FLS.

Figure 27 shows an important stabilization in the trajectory of the AMV when FBCO-IT3FLS is implemented, this is due to a better evaluation of the uncertainty, in this case, it is visualized in fuzzy control through adding disturbance in the model. Another visualization on the results is shown in Figure 28.

In Figure 28 an extraction of the trajectory with the goal of improving the visualization in the results, where the original BCO (a) and the proposed methodology (FBCO-IT3FLS) illustrate a stabilization on the trajectory for the AMR. When FBCO-IT3FLS is used, with disturbance in the model, the desired trajectory (red color) compared to the result of the trajectory with the proposal (blue color) allow a lower separation between the lines.

Table 3 shows an analysis of the α and β parameter values to find for each Fuzzy BCO algorithm when the disturbance is added.

Table 3 shows the averages of the values to α and β parameters for each fuzzy BCO algorithm, the range to find for the β is of [2.760, 3.558] and the range to find for the α is of [0.541,0.803]; the best value in the minimization of the error with FBCO-IT3FLS the α and β values are **0.488** and **2.602**, respectively. It is important to mention, when the FBCO-IT3FLS is implemented the α and β values are different comparing with FBCO-T1FLS that the values are constantly repeated for example, **3.625** and **0.818** to α and β. With FBCO-IT2FLS and FBCO-GT2FLS, six different values are found to α and β parameters. However, with FBCO-IT3FLS each experiment allows finding a different value to α and β parameters, because the proposed fuzzy BCO is evaluated with greater precision and better analyzes the uncertainty.

## 6. Statistical Test

A parametric statistical test is analyzed to check the performance and each type of fuzzy set. In order to corroborate all the experimentation carried out with the proposed method, several statistical tests are developed. The parameters of the statistical test are: confidence level 95%, alpha 0.05, sample size is random sample of 30 and the critical value is −1.645. Where:

1 = FBCO-IT3FLS

2 = Original BCO or FBCO-T1FLS or FBCO-IT2FLS or FBCO-GT2FLS

The hypotheses would be as follows: Ho: 1 ≥ 2 and Ha: 1<2 Claim. Thus, an acceptance of Ho indicates that there exists sufficient evidence for values below the critical value Z of −1.645 then Ha is rejected. The Equation (43) shows the z-test:

(43) $$Z=\frac{\left({\bar{X}}_{1}-{\bar{X}}_{2}\right)-\left(\mu_{1}-\mu_{2}\right)}{\sigma_{{\bar{X}}_{1}-{\bar{X}}_{2}}}$$

Table 4 shows the FBCO-IT3FLS results without perturbation which represents our 1 and we perform a statistical test for each of the 4 methods (Original BCO, FBCO-T1FLS, FBCO-IT2FLS and FBCO-GT2FLS) which will represent 2 in each of the statistical tests.

The same methodology is applied in Table 5 where 4 statistical tests are performed but in comparison with the FBCO-IT3FLS results with perturbation. The claim is that the FBCO-IT3FLS method has lower results compared to the Original BCO algorithm.

Based on results on Table 4 and Table 5 for the proposed method (FBCO-IT3FLS), the analysis is that in all cases the proposal has significant evidence when compared to the 4 methods of the literature, when disturbance is added in the model.

## 7. Conclusions

The bio-inspired algorithms are used in different types of problems. To find the appropriate parameter settings for each algorithm could be very complicated. For this reason, is necessary to explore the performance of the algorithm to find the optimal values in the parameters. In this paper, a study and analysis to determine the influence that the α and β values present on the performance of BCO applied to FC is presented. In addition, the optimal design of the structure and fuzzy rules to the FBCO-IT3FLS are obtained.

According to the results in Section 5, we find that BCO is an excellent technique for the design and optimization of the FC, because the study case without applying perturbation presents stabilization (See Table 2), but when the FLC is used with level of noise, the stabilization is better with the proposed method (see Figure 27 and Figure 28). This is because IT3FLS allows a better handling of the uncertainty and the perturbations is minimized. The statistical test reflected acceptance of the Ho when disturbance is added in the model and a FBCO-IT3FLS is implemented to find the appropriate α and β values in BCO (see Table 4 and Table 5).

When an IT3FLS is implemented to search for the values to α and β parameters the diversity in the values is wider when compared to Original BCO with three different values in these parameters. When the uncertainty is considered in the evaluation, this allows the values found by the BCO algorithm in hybridization with each fuzzy set to be more diverse (See Table 3).

The complexity of the proposed FBCO-IT3FLS algorithm is analyzed with a fast convergence on the results (See Figure 24), and when perturbation is added in the model, better results are obtained (See Figure 25 and Figure 26). Although several mathematical calculations are performed in each evaluation for the IT3FMs, the computational time is very similar to the GT2MFs, and the results are very significant (See Section 6).

Some important limitations that should be analyzed are the computational time—in this first phase, many evaluations are executed when an IT3FLS is used—and being able to count on the appropriate hardware for real-time simulation would be an efficient way to check the robustness of an IT3FLS in uncertain environments.

Some interesting extensions to this research are: optimizing fuzzy controllers of the Interval Type-2 FLC, Generalized FLC and Interval Type-3 FLC by applying several perturbations on the model of the plant; implementing this proposed methodology in the optimization of Benchmark Functions and, mainly, to use this proposal of the implementation of IT3FLS to find optimal parameters of bio-inspired algorithms in nature.

## Figures and Tables

**Table 1:** Simulation errors for each Fuzzy BCO algorithm without disturbance in the model.

Performance Inde×		Methods				
		Original BCO	FBCO-T1FLS	FBCO-IT2FLS	FBCO-GT2FLS	FBCO-IT3FLS
**ITAE**		1.94 × 10^+3^	1.97 × 10^+3^	1.95 × 10^+3^	1.96 × 10^+3^	1.96 × 10^+3^
ITSE		7.81 × 10^+2^	8.03 × 10^+2^	7.80 × 10^+2^	7.91 × 10^+2^	7.87 × 10^+2^
IAE		3.92 × 10^+1^	3.98 × 10^+1^	3.94 × 10^+1^	3.96 × 10^+1^	3.96 × 10^+1^
ISE		1.59 × 10^+1^	1.63 × 10^+1^	1.59 × 10^+1^	1.60 × 10^+1^	1.60 × 10^+1^
MSE		3.27 × 10^0^	1.31 × 10^0^	**9.83 × 10^−1^**	1.46 × 10^0^	1.19 × 10^0^
RMSE		1.60 × 10^0^	1.75 × 10^0^	1.73 × 10^0^	1.52 × 10^0^	1.67 × 10^0^
MSE	Std.	2.08 × 10^0^	2.10 × 10^0^	1.15 × 10^0^	3.46 × 10^0^	1.79 × 10^0^
	Best	8.99 × 10^−3^	1.03 × 10^−2^	1.03 × 10^−2^	1.03 × 10^−2^	1.34 × 10^−2^
	Worst	1.05 × 10^+1^	7.72 × 10^0^	3.77 × 10^0^	1.82 × 10^+1^	6.58 × 10^0^
Beta		3 (Fixed)	3.625	2.599	2.478	2.734
Alpha		0.5 (Fixed)	0.818	0.466	0.456	0.555

**Table 2:** Simulation errors for each Fuzzy BCO algorithm with perturbation.

Performance Inde×		Methods				
		Original BCO	FBCO-T1FLS	FBCO-IT2FLS	FBCO-GT2FLS	FBCO-IT3FLS
ITAE		1.96 × 10^+3^	1.97 × 10^+3^	1.97 × 10^+3^	1.96 × 10^+3^	1.95 × 10^+3^
ITSE		7.93 × 10^+2^	7.96 × 10^+2^	7.94 × 10^+2^	7.88 × 10^+2^	7.91 × 10^+2^
IAE		3.96 × 10^+1^	3.97 × 10^+1^	3.97 × 10^+1^	3.96 × 10^+1^	3.94 × 10^+1^
ISE		1.16 × 10^+1^	1.61 × 10^+1^	1.61 × 10^+1^	1.60 × 10^+1^	1.60 × 10^+1^
MSE		9.93 × 10^−1^	9.34 × 10^−1^	2.16 × 10^0^	1.40 × 10^0^	1.00 × 10^0^
RMSE		1.54 × 10^0^	1.33 × 10^0^	1.72 × 10^0^	1.59 × 10^0^	1.53 × 10^0^
MSE	Std.	1.78 × 10^0^	1.83 × 10^0^	4.65 × 10^0^	3.35 × 10^0^	1.44 × 10^0^
	Best	7.53 × 10^−3^	1.03 × 10^−2^	1.03 × 10^−2^	1.03 × 10^−2^	**2.61 × 10^−3^**
	Worst	6.00 × 10^0^	7.72 × 10^0^	1.83 × 10^+1^	1.82 × 10^+1^	4.46 × 10^0^
Beta		3 (Fixed)	3.625	2.640	2.568	2.602
Alpha		0.5 (Fixed)	0.818	0.530	0.486	0.487

**Table 3:** Results of the α and β values.

Experiment	FBCO-T1FLS		FBCO-IT2FLS		FBCO-GT2FLS		FBCO-IT3FLS	
	β	α	β	α	β	α	β	α
**1**	3.626	0.818	2.812	0.570	3.021	0.456	2.645	0.510
**2**	3.287	0.742	2.604	0.469	2.973	0.487	2.571	0.472
**3**	3.625	0.818	2.623	0.476	2.881	0.505	3.277	0.831
**4**	3.625	0.818	2.599	0.466	2.881	0.505	2.572	0.472
**5**	3.287	0.742	2.941	0.643	2.973	0.487	3.277	0.832
**6**	3.625	0.818	2.599	0.465	2.973	0.487	2.816	0.599
**7**	3.625	0.818	3.010	0.635	2.973	0.487	2.572	0.472
**8**	3.625	0.818	2.640	0.530	3.021	0.456	2.816	0.599
**9**	3.287	0.742	2.640	0.530	3.021	0.456	3.277	0.832
**10**	3.287	0.742	2.640	0.530	2.881	0.505	2.571	0.472
**11**	**3.625**	**0.818**	2.640	0.530	2.881	0.505	2.570	0.472
**12**	3.625	0.818	2.640	0.530	2.973	0.487	2.572	0.472
**13**	3.625	0.818	2.640	0.530	2.881	0.505	2.624	0.499
**14**	3.625	0.818	2.640	0.530	3.021	0.456	2.794	0.588
**15**	3.625	0.818	2.640	0.530	2.881	0.505	2.642	0.509
**16**	3.625	0.818	2.640	0.530	3.021	0.456	2.572	0.472
**17**	3.625	0.818	3.010	0.635	2.881	0.505	2.906	0.645
**18**	3.625	0.818	2.640	0.530	2.973	0.487	2.572	0.472
**19**	3.625	0.818	3.010	0.635	2.973	0.487	2.570	0.471
**20**	3.287	0.742	2.640	0.530	2.881	0.505	2.655	0.521
**21**	3.625	0.818	2.640	0.530	2.973	0.487	**2.602**	**0.488**
**22**	3.625	0.818	2.640	0.530	2.881	0.505	2.593	0.483
**23**	3.625	0.818	2.640	0.530	2.881	0.505	2.793	0.587
**24**	3.625	0.818	2.640	0.530	**2.973**	**0.487**	2.629	0.502
**25**	3.625	0.818	**2.640**	**0.530**	2.881	0.505	2.774	0.578
**26**	3.625	0.818	2.640	0.530	3.021	0.456	2.644	0.510
**27**	3.625	0.818	2.640	0.530	3.021	0.456	2.605	0.489
**28**	3.625	0.818	2.640	0.530	3.021	0.456	3.277	0.832
**29**	3.625	0.818	3.010	0.635	2.973	0.487	2.718	0.548
**30**	3.287	0.742	2.640	0.530	2.973	0.487	3.277	0.832
**AVERAGE**	3.558	0.803	2.703	0.541	2.952	0.485	2.760	0.569

**Table 4:** Statistical test results for proposal FBCO-IT3FLS without Perturbation.

Method	FBCO-IT3FLS	Original BCO	FBCO-T1FLS	FBCO-IT2FLS	FBCO-GT2FLS
**Minimun**	1.34 ×10^−2^	8.99 × 10^−3^	1.03 × 10^−2^	1.03 × 10^−2^	1.03 × 10^−2^
**Maximum**	6.58 × 10^0^	1.05 × 10^+1^	7.72 × 10^0^	3.77 × 10^0^	1.82 × 10^+1^
**Average**	1.19 × 10^0^	1.12 × 10^0^	1.31 × 10^0^	9.83 × 10^−1^	1.46 × 10^0^
**Std.**	1.79 × 10^0^	2.08 × 10^0^	2.10 × 10^0^	1.15 × 10^0^	3.46 × 10^0^
**Z Value**		−4.6307	−1.4274	−1.4274	−1.4274
**Evidence**		Significative	Not Significative	Not Significative	Not Significative

**Table 5:** Statistical test results for proposal FBCO-IT3FLS with Perturbation.

Method	FBCO-IT3FLS	Original BCO	FBCO-T1FLS	FBCO-IT2FLS	FBCO-GT2FLS
**Minimun**	2.61 × 10^−3^	7.53 × 10^−3^	1.03 × 10^−2^	1.03 × 10^−2^	1.03 × 10^−2^
**Maximum**	4.46 × 10^0^	6.00 × 10^0^	7.72 × 10^0^	1.83 × 10^+1^	1.82 × 10^+1^
**Average**	1.00 × 10^0^	9.93 × 10^−1^	9.34 × 10^−1^	2.16 × 10^0^	1.40 × 10^0^
**Std.**	1.44 × 10^0^	1.78 × 10^0^	1.83 × 10^0^	4.65 × 10^0^	3.35 × 10^0^
**Z Value**		−1.8109	−2.1111	−2.1111	−2.1111
**Evidence**		Significative	Significative	Significative	Significative
